# A Structured Benefit-Risk Assessment Operating Model for Investigational Medicinal Products in the Pharmaceutical Industry

**DOI:** 10.1007/s43441-023-00508-2

**Published:** 2023-04-01

**Authors:** Tim Sullivan, Gyorgy Zorenyi, Jane Feron, Meredith Smith, Magnus Nord

**Affiliations:** 1 Global Patient Safety BioPharmaceuticals, Chief Medical Office, R&D, AstraZeneca, 200 Orchard Ridge Drive, Gaithersburg, MD 20878 USA; 2 Global Patient Safety Oncology, Chief Medical Office, R&D, AstraZeneca, Cambridge, UK; 3 Global Patient Safety, Epidemiology and Risk Management, Chief Medical Office, R&D, AstraZeneca, Cambridge, UK; 4 Formerly of Global Patient Safety, Epidemiology and Risk Management, Chief Medical Office, R&D, Alexion-AstraZeneca Rare Disease, Boston, MA USA; 5 Global Patient Safety BioPharmaceuticals, Chief Medical Office, R&D, AstraZeneca, Gothenburg, Sweden

**Keywords:** Benefit-risk, Structured benefit-risk, Health authorities, Key clinical benefits, Key safety risks

## Abstract

Robust and transparent formal benefit-risk (BR) analyses for medicinal products represent a means to better understand the appropriate use of medicinal products, and to maximize their value to prescribers and patients. Despite regulatory and social imperatives to conduct structured BR (sBR) assessments, and the availability of a plethora of methodological tools, there exists large variability in the uptake and execution of sBR assessments among pharmaceutical companies. As such, in this paper we present an sBR assessment framework developed and implemented within a large global pharmaceutical company that aims to guide the systematic assessment of BR across the continuum of drug development activities, from first-time-in-human studies through to regulatory submission. We define and emphasize the concepts of Key Clinical Benefits and Key Safety Risks as the foundation for BR analysis. Furthermore, we define and foundationally employ the concepts of sBR and a Core Company BR position as the key elements for our BR framework. We outline 3 simple stages for how to perform the fundamentals of an sBR analysis, along with an emphasis on the weighting of Key Clinical Benefits and Key Safety Risks, and a focus on any surrounding uncertainties. Additionally, we clarify existing definitions to differentiate descriptive, semi-quantitative, and fully quantitative BR methodologies. By presenting our framework, we wish to stimulate productive conversation between industry peers and health authorities regarding best practice in the BR field. This paper may also help facilitate the pragmatic implementation of sBR methodologies for organizations without an established framework for such assessments.

## Background

The history of formal benefit-risk (BR) assessment in the pharmaceutical industry dates back to the 1990s, in particular, the 1998 CIOMS Working Group IV (Benefit-Risk Balance for Marketed Drugs: Evaluating Safety Signals). This was followed in the 2000s by the Benefit-Risk Action Team (BRAT), a collaborative project on BR evaluation sponsored by Pharmaceutical Research and Manufacturers of America (PhRMA), as well as several formal regulatory initiatives, e.g., the European Medicines Agency (EMA’s) BR methodology workstream, the United States Food and Drug Administration (FDA’s) BR framework, the International Council for Harmonization M4E initiative, and the Innovative Medicine Initiative’s (IMI) Pharmacoepidemiological Research on Outcomes of Therapeutics in a European Consortium (PROTECT) project.

Collectively, these initiatives resulted in a variety of structured BR (sBR) frameworks. The use of structured frameworks enabled a more systematic, transparent, rigorous, and flexible approach to BR assessment, one that was able to accommodate a range of analytic methods, including quantitative approaches when appropriate [1]. Since then, major regulators, such as EMA and FDA, have implemented mandatory sBR approaches for reviewer teams to use when assessing product licensing applications [2–4]. These requirements have been adopted to varying degrees across the pharmaceutical industry. A recent survey [5] documented that a large majority of pharmaceutical companies do perform some type of sBR assessment. A second, subsequent survey [6] demonstrated similar findings and further emphasized a trend towards companies initiating BR assessments earlier in drug development. Other recent industry case studies have illustrated how BR assessment frameworks can be effectively integrated into an organizational context [7, 8].

Despite the foregoing efforts, in our view numerous questions remain that impede widespread uptake and adoption of sBR assessment within the pharmaceutical industry [9]. While the combined efforts of these various BR initiatives have identified a large set of BR methodologies that are suitable for use in the pharmaceutical industry, there remains a lack of consensus regarding a best practice framework to guide selection and application of such methods [10, 11].

## Conceptual Highlights of the AstraZeneca sBR Framework Initiative

Despite the substantial progress in BR assessment over the past 20 + years, a recent survey observed that concerns over the value proposition of more formal and quantitative BR assessments persist among some industry stakeholders. Several barriers have been cited, ranging from organizational inertia to lack of clear BR definitions, to the fact that all health authorities do not yet mandate the use of a specific sBR or specific type of quantitative BR assessments [5].

At AstraZeneca, our starting position was that the assessment of BR for a medicinal product represents the apex deliverable for any pharmaceutical research and development (R&D) organization. As such, the development of concise, transparent, and compelling formal BR assessments is operationally defined as a value-adding process. Examples of how such assessments are value adding include their ability to inform next phase internal investment decisions, to ascertain early in the program that the right data are collected in later clinical trials, to help summarize due diligence activities, to communicate a product’s BR profile to senior management and cross-departmentally, and for submitting a product for regulatory approval [4, 12].

Furthermore, given the necessity and importance of these formal BR assessments, we proceeded from the perspective that they should always be done in a structured way [11]. Indeed, we attach such importance to the concept of structure that we have internalized the “sBR” term and produced a working definition for the term (further below).

In a further step to educate stakeholders, we developed a simple 3-step working definition on how to perform an sBR assessment:

1. *Agree on the definitions, descriptions, and facts*: Key Clinical Benefits and Key Safety Risks are the factors comprising an sBR analysis. These terms are discussed further below.
2. *Agree on the relative importance of, and any uncertainty surrounding, the Key Clinical Benefits and Key Safety Risks*: When we discuss relative importance, we mean the concept of weighting of Key Clinical Benefits and Key Safety Risks; in other words, ranking (and sometimes quantifying) the medical importance of these variables. When we discuss uncertainty, we mean missing information or the potential for various biases, which make interpretation difficult.
3. *Produce a concise and clear (i.e., with a standalone quality) BR assessment*: Use a situationally appropriate and widely recognized methodology with the output being a 1–2-page BR summary expressing the company core BR position. The intent is that a given medical reviewer can quickly and efficiently understand the company’s BR narrative for a given product.

We have also reinforced the concept of a Core Company BR position, which is analogous to the concept of a Core Company Data Sheet (CCDS), but in this case used in guiding BR communications both internally and externally (Note that the concept of a Core Company BR position was introduced initially by Smith et al. [7]).

Additionally, we have chosen the tripartite categorization of BR methodologies (descriptive, semi-quantitative, and fully quantitative) and provided working definitions of each. We have also created an organically progressive methodological framework to guide project teams in sBR assessments, from the beginning of clinical development through to submission and the post-marketed setting.

One final observation of pivotal importance to the organizational success of implementing an sBR procedural framework is the buy-in of all involved stakeholders. Benefit-risk assessment is, by definition, a highly cross-functional undertaking. Indeed, a recent survey [6] has shown that BR work in pharmaceutical companies is shared across a variety of functions, in particular clinical development, patient safety, biostatistics, regulatory and epidemiology functions. Any framework developed without the support of any of these key functions is unlikely to be fully effective or adopted. For companies beginning early phase sBR assessments, the contributor list should also include experts in early development and preclinical medicine.

## Details of the AstraZeneca sBR Framework Refresh

For context, until this time AstraZeneca had been using the PhRMA BRAT model as a framework for BR assessments, which were generally limited to the use of forest plots generated only in late phase development. With accumulating experience, our view was that a broader range of methodological options were needed, and that BR assessments should commence earlier in development.

### Defining sBR

We have defined five principles which we believe are fundamental to the definition of structured BR, per se.

1. *A highly succinct and standalone presentation*. We recommend the Core Company BR position is limited to a single page output that will serve as the core position for all related BR synopses. The intention is that any internal or external medical reviewer can immediately and clearly understand the basics of our core position on BR without any supplementary material.
2. *A highly concise number of specifically defined benefits and risks that minimize overlap and duplication with one another*. Via careful prioritization and weighting, we generally aim for no more than 2–3 Key Clinical Benefits and no more than 6–8 Key Safety Risks to be included, all of which are as precisely defined and mutually exclusive as possible. The goal is to demonstrate that, as experts on our products, we are able to clearly delineate the most important benefits and risks.
3. *Rigorous assessment of the clinical importance of endpoints and any uncertainties surrounding them*. All benefits and risks should be strictly assessed by the “feel, function, and survive” rubric espoused by the FDA [13] and the European Network for Health Technology Assessment [14]. For example, a clinically-based secondary endpoint (e.g., clinical symptoms) may sometimes outweigh a lab-measured primary endpoint in sBR analysis, as the lab measure may not clearly translate into a specific clinical effect. Additionally, if any Key Clinical Benefits or Key Safety Risks are surrounded by uncertainty (e.g., missing data, potential biases), it is critical to incorporate and contextualize this when choosing a presentation.
4. *Adherence to widely recognized BR framework(s)*. While a large variety of potential BR methodologies exist, a limited number have either been developed or sanctioned by major health authorities [15–18].
5. *sBR assessments should be conducted at defined milestones, beginning early in development*. In our view, sBR analysis using such methodologies should begin around the time of first-time-in-human (FTIH) studies and be periodically reviewed at the time of critical developmental milestones, such as investment decisions; when it is more practical to change clinical development plans, if needed [6].

### Defining Key Clinical Benefits and Key Safety Risks

A concise number of Key Clinical Benefits (i.e., favorable effects) must be determined for any sBR analysis, and these should be consistent with the primary and secondary efficacy endpoints of the pivotal clinical studies. While the primary efficacy endpoint will often be the most important Key Clinical Benefit for sBR assessment, in certain situations, the secondary endpoints might be more important (for example, when the primary endpoint is a proxy lab measure of uncertain clinical impact as opposed to a secondary endpoint representing a clinical outcome, such as symptom relief). Of primary importance is the clear demonstration of clinically meaningful outcomes for patients, defined by the FDA in terms of how a patient “feels, functions, and survives” [13]. In certain situations, non-endpoint variables may also be considered Key Clinical Benefits; for example, increased quality of life, or degree of adherence to a distinct route of administration. If such variables emerge as key benefits, they may be included as secondary endpoints in future protocols.

Key Safety Risks are unfavorable effects that are important due to their potential impact on patients and the approvability or clinical use of the product (e.g., in terms of morbidity, mortality, hospitalizations, compliance, etc.). As safety data are typically characterized descriptively (i.e., not usually by use of inferential statistics) in most registrational studies, determining Key Safety Risks can be a challenge, requiring careful medical judgment. The number of Key Safety Risks is typically greater than the number of Key Clinical Benefits, and may include either identified (i.e., causally related) and/or potential (i.e., not yet determined to be causally related) risks [19].

While Key Safety Risks generally overlap with Important Risks from the Risk Management Plan (RMP; as defined by ‘Guideline on good pharmacovigilance practices’ Module V revision 2), full correlation is not required, as the criteria are not the same. In other words, it is possible to have a Key Safety Risk that is not an RMP Important Risk and vice versa. For example, a class effect label warning to periodically assess the need for vitamin supplementation could be in the RMP, as clinical action by prescribers is required in this instance; however, it would not be a Key Safety Risk since, as a well-recognized and manageable class effect, it is unlikely to affect the approvability or clinical use of the product.

It is considered best practice to ensure that Key Clinical Benefits and Key Safety Risks are precisely defined and mutually exclusive [4, 11, 20]. This helps to avoid double-counting events [20]; for example, counting cerebral hemorrhage as both an efficacy endpoint (e.g., major adverse cardiovascular event) and a safety endpoint (e.g., serious adverse event of severe bleeding). This effort to avoid double-counting of events should be focused on defining precise and mutually exclusive definitions for key benefits and risks in the construction of a value tree. While there is a general need for being precise and mutually exclusive in defining the Key Clinical Benefits and Key Safety Risks, it is most important for fully quantitative (i.e., net clinical benefit) analyses to avoid making inaccurate calculations. To support the definition of Key Safety Risks, we have defined criteria, listed in Table 1.

**Table 1:** Criteria for defining Key Safety Risks

*Key safety risks*
1. Signal strength and likelihood of causality
2. Seriousness and clinical impact of the safety risk, were it to occur
3. Manageability of the safety risk: Is it predictable, reversible, or treatable
4. Patient-centric aspects of the safety risk: Is the safety risk particularly onerous to the target population
5. Differentiation potential of the safety risk: Is there a particular need to differentiate from competitors or standard of care

### Defining the Use of Weighting in sBR

In simple terms, we define weighting in sBR analysis as acknowledging that some benefits and risks are more important than others. While this concept may be implicit (i.e., since people informally do it in their mind), the practical application of attaching discrete numerical values reflecting the relative importance of Key Clinical Benefits and Key Safety Risks is historically less common within the pharmaceutical industry [1], and anecdotally represented the biggest hurdle for our own staff.

Weighting of Key Clinical Benefits and Key Safety Risks is most important for fully quantitative analyses, including Multi-Criteria Decision Analysis (MCDA) [11, 21]. In such analyses, discrete numerical values are attached to each Key Clinical Benefit and Key Safety Risk, using the weighting (i.e., relative importance) and frequency assigned to each, and then the numerical values of the summed Key Clinical Benefits are compared to the value of the summed Key Safety Risks. We also believe that some sort of more limited weighting exercise is useful even if fully quantitative methods are not used, as recognizing the relative importance of Key Clinical Benefits and Key Safety Risks helps focus developmental priorities.

There are a variety of weighting systems available but, to our knowledge, there is no clear consensus on which is best [22]. Regardless of the methodology chosen, an obvious challenge with any weighting system is one of minimizing subjectivity of the raters. We believe that there is value in sponsor project teams performing a weighting exercise, at least for the purposes of internal decision-making. However, we recognize that external stakeholders may want to see weighting performed by more independent parties, such as key opinion leaders or patient groups [11, 23].

As detailed further below, we employ a rudimentary weighting exercise around the Phase II timepoint since we believe the relative prioritization of key benefits and risks helps focus developmental decision-making. The results of this weighting exercise are then carried over into subsequent semi-quantitative or quantitative analyses.

### Defining sBR Categories

Historically, assessments of BR in the pharmaceutical industry have often been descriptive in nature. With increasing experience, there has been an effort to implement fully quantitative analysis [1]. While several BR systems use these two categories to describe possible frameworks, we believe the addition of a third category (semi-quantitative) has value. Our internal definitions are as follow:

1. Descriptive BR analysis: Primarily text-based, with limited focus on any quantitative analyses. These are the type of analyses that are more appropriate to serve as a Core Company BR position in early development when data are limited. These are also frequently appropriate for communicating BR in clinical trial documents, such as an investigator’s brochure or a clinical study protocol. In certain situations, such as the submission of a medicinal product with extremely limited or difficult to interpret data, a descriptive BR analysis may still be appropriate at the time of regulatory submission.
2. Semi-quantitative BR analysis: A heavier focus on quantitative analyses, such as reporting hazard or risk ratios in the form of effects tables or forest plots, etc., which may still be accompanied by some degree of explanatory context. It is our view that semi-quantitative analysis should be the base case approach for most, if not all, later phase products, particularly those ready for regulatory submission.
3. Fully quantitative BR analysis: A clear focus on assigning a specific quantitative value to each Key Clinical Benefit and each Key Safety Risk to achieve numerical values that, when compared, represent net clinical benefit. It is our view, and that of certain health authorities [24], that fully quantitative BR assessments may sometimes offer value in more challenging or complex scenarios where semi-quantitative analyses are insufficient. It should be noted that fully quantitative analyses by definition require a formal and detailed weighting exercise.

### Definition and Use of a Core Company BR Position

Health authorities and the pharmaceutical industry are familiar with the concept of a CCDS, which represents the company’s views on the core medical-scientific information on efficacy, safety, etc., which form the basis for local labels and prescribing information [7].

We now use a similar concept to the CCDS at AstraZeneca with respect to sBR assessments, which are referred to as the Core Company BR position. The Core Company BR position (CBR) is very concise (i.e., 1–2 pages) and reflects the sBR assessment for a given product. All internal or external communications associated with BR must be based on, and in line with, the CBR position. This becomes a practical matter in clinical study documents or periodic safety reports, where in-text descriptive statements regarding BR may be more appropriate than the highly concise Core Company BR position. Notably, the Core Company BR position is dynamic and requires updating if new important data affecting the overall BR profile are received.

### Developmental Milestone-Specific sBR Methodology Framework

A defining principle of sBR is the need to begin BR analysis early in development. Our anecdotal experience is that waiting until the time of submission results in project teams conducting BR analysis in a reactive and less prepared manner than if they had started the process earlier. By starting sBR analysis at the time of first-time-in-human (FTiH) studies, familiarity with the principles of sBR are established early, arguably resulting in a more robust and efficient sBR assessment at the time of submission. Furthermore, it allows for emerging key risks and benefits to influence which data are collected later in the development program, as well as which endpoints are selected for assessment. Notably, the FDA has recently stated the importance of BR discussion at the end of Phase II meetings, which presumes some degree of early work on BR [13].

**Figure 1:**
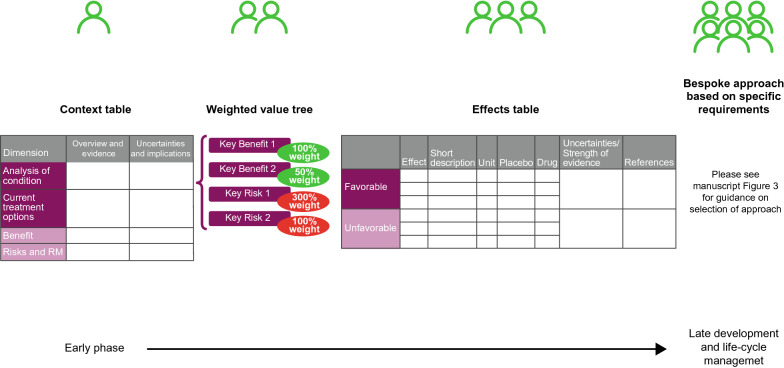
Developmental milestone-specific sBR methodology framework. *NNH* Number needed to harm, *NNT* number needed to treat, *RM* risk management, *sBR* structured benefit-risk.

At AstraZeneca, the Global Patient Safety group administers safety strategies for investigational products, with a pronounced focus at key developmental milestones, such as FTIH studies and certain investment decisions (i.e., at Phase IIb, Phase III, and submission) [25]. These milestones represent the most efficient opportunity to leverage input into clinical development plans. Thus, our new sBR process was established around these same milestones (Fig. 1).

The AstraZeneca sBR methodology framework is established with a focus on the most recognized BR frameworks, particularly those methods which have been generated, or in some way endorsed, by health authorities. The overall strategy is to start with a light touch at the beginning of clinical development and incrementally establish the CBR position as the project progresses. Our recommended methodologies are chosen and sequenced in such a way that by the time of regulatory submission, project teams will already have several well-recognized sBR analyses of increasing complexity in place and will be better prepared to perform a more complex sBR analysis, if needed.

#### FTIH Milestone

Our recommended methodology for the FTIH milestone is a modified version of the FDA BR Framework [13], which we refer to as a context table (Table 2). Our observation is that early in development, it is not always possible to define Key Clinical Benefits and Key Safety Risks. Instead, we focus on contextual information relating to the treatment space, combined with high level overviews of the emerging medical-scientific evidence package. Generally, this information is already established across several internal sources (and thus does not require much resource to produce), but had historically not been collated together in one place to begin a BR narrative. To best utilize this modified FDA framework, we have developed specific instructions for its use (Appendix 1).

**Table 2:** Modified FDA BR framework (context table).

	Overview and evidence	Uncertainties and implications
Analysis of condition		
Current treatment options		
Potential clinical benefits		
Potential safety risks		

*BR* Benefit-risk, *FDA* the United States Food and Drug Administration

#### Phase IIb Investment Decision Milestone

**Figure 2:**
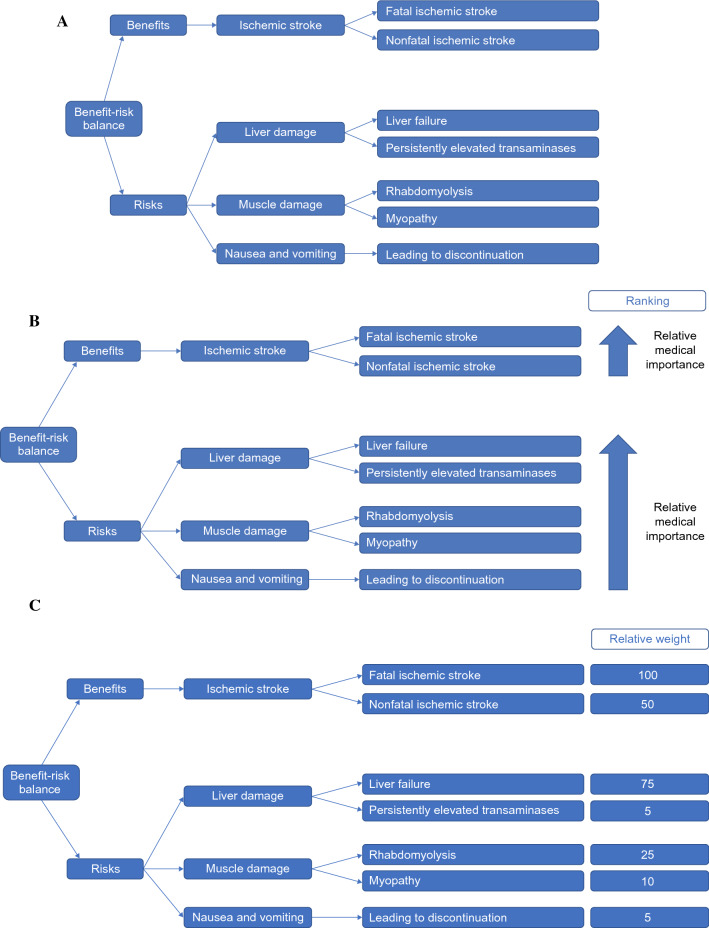
**A** Simple value tree. Example of a simple value tree. Content is fictive and for illustrative purposes only. Figure adapted from Fig. 2 in Coplan et al. [26]. **B** Weighted value tree. Example of a weighted value tree. Content is fictive and for illustrative purposes only. Figure adapted from Fig. 2 in Coplan et al. [26]. **C** Complete weighted value tree. Example of a complete weighted value tree. Content is fictive and for illustrative purposes only, and is not intended to represent actual medical judgments. Figure adapted from Fig. 2 in Coplan et al. [26].

By the time of the Phase IIb investment decision we expect Key Clinical Benefits and Key Safety Risks will usually have been identified and represented in the form of a value tree (Fig. 2A). This is the simplest and most fundamental sBR methodology [26, 27], as its production is the first step towards any more complex analysis.

An additional recommendation at this milestone is the introduction of weighting, which is represented in the form of a weighted value tree (Fig. 2B). The basic requirement is to rearrange the value tree based on a simple ordinal ranking of the relative medical importance of individual Key Clinical Benefits and Key Safety Risks. It should be reiterated that known or estimated frequencies of Key Safety Risks do not factor into the weighting process; instead, this exercise is focused solely on relative medical importance. When performing such ordinal ranking, it is helpful to think in terms of the single occurrence of a Key Clinical Benefit or Key Safety Risk. For example, does the occurrence of a given Key Clinical Benefit in a single patient weigh more or less in terms of medical importance than the single occurrence of a given Key Safety Risk in a single patient.

For products with more complicated BR profiles, and a potential need for fully quantitative sBR analysis moving forward, a more detailed form of weighting for the value tree other than ordinal ranking is recommended. Our choice of weighting methodology consists of a simple Delphi process [28], where the single most important Key Clinical Benefit can be assigned an arbitrary value of ‘100’, and all other Key Clinical Benefits and Key Safety Risks are assigned values relative to that anchor point (Fig. 2C). Instructions to this process are included in “Appendix 2” section.

Finally, the use of basic, ordinal-weighted value trees can be a very fit-for-purpose way to compare and contrast the high-level BR profile for products with multiple indications.

#### Phase III Investment Decision Milestone

By the time pivotal trials are underway, certain projects may have enough data to fill out an effects table. The effects table is a commonly used tool which is part of the EMA’s PROaCT-URL framework [3] (Table 3), which contains a list of Key Clinical Benefits and Key Safety Risks with their observed (or estimated) frequencies, along with in-text descriptions of any relevant context, particularly any important uncertainties. In cases where data from previous phases of clinical development are limited (e.g., accelerated development programs in high unmet medical need; indications for rare diseases or underserved oncology indications), weighted value trees are considered an alternative methodology, with the Effects Table to be completed later as possible during Phase III.

**Table 3:** EMA effects table.

	Effect	Short description	Unit	Placebo	Vandetanib	Uncertainties/strength of evidence	References
Favorable	PFS (HR)	From randomization to progression or death (blinded independent review)	N/A	1	0.5; 95% CI (0.3, 0.7)	Large effect in overall population. Consistent and significant effect on PFS but not OS (too early?)	See Discussion on clinical efficacy
	PFS (median)	Weibull model	Mo	19.3	30.5	Only a very low number of patients with definitive RET mutation negative status at baseline. Lower efficacy?	Single-arm study in RET negative patients post-approval
	ORR	Proportion of complete or partial responders (≥ 30% decrease unidimensional) RECIST	%	13	45	No clear effect on PRO/QoL (missing data)	See Discussion on clinical efficacy
Unfavorable	Diarrhea Grade 3–4	Increase of ≥ 7 stools per day over baseline; incontinence; life-threatening	%	2.0	10.8	Duration of follow-up in the pivotal study is short vs the need for long duration of treatment	Risk of dehydration and renal/cardiac risks (see SmPC 4.4)
	QTc related events Grade 3–4	QTc > 0.50 s; life-threatening; Torsade de pointes	%	1.0	13.4	Risk of developing further major cardiac SAEs, including Torsade de pointes	Restrict to symptomatic and aggressive disease (see SmPC 4.1)
	Infections Grade 3–4	IV antibiotic, antifungal, or antiviral intervention indicated; life-threatening	%	36.4	49.8		Explore lower dose (see Table 20- summary of the RMP)

Example of an effects Table [3]

*CI* confidence interval, *EMA* European Medicines Agency, *HR* hazard ratio, *IV* intravenous, *Mo* months, *N/A* not applicable, *ORR* objective response rate, *OS* overall survival, *PFS* progression-free survival, *PRO* patient-reported outcome, *QoL* quality of life, *RECIST* Response Evaluation Criteria in Solid Tumors, *RET* “rearranged during transfection” gene, *RMP* Risk Management Plan, *SAE* serious adverse event, *SmPC* summary of product characteristics

#### Submission Investment Decision Milestone

**Figure 3:**
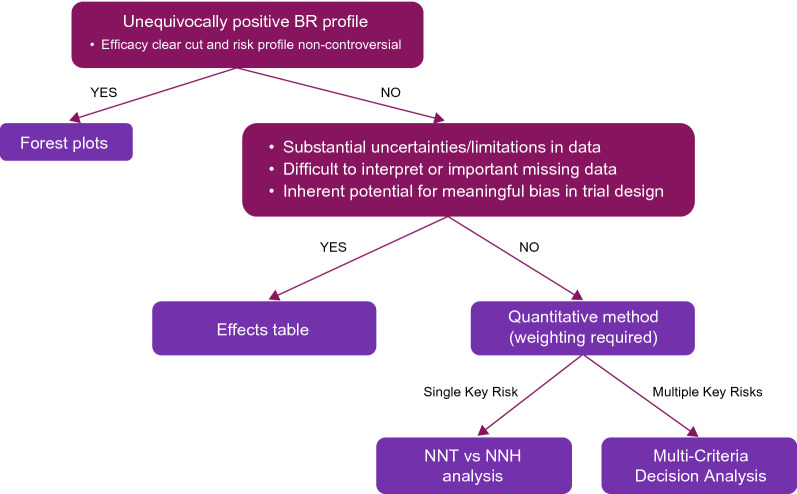
BR methodology choice for regulatory submission. *BR* Benefit-risk, *NNH* number needed to harm, *NNT* number needed to treat.

At the time of submission for marketing approval, our view is that a semi-quantitative Core Company BR position should generally be the base case methodology. At AstraZeneca, recommended semi-quantitative methodologies include an effects table or a forest plot. Fully quantitative options, such as MCDA or number needed to treat (NNT), are recommended for products with more complex BR scenarios. To aid decision-making at this point, we have derived an algorithm based on certain characteristics unique to each project (Fig. 3). Another algorithm, which we find useful, has also been developed independently [11]. Some scenarios may benefit from a combination of these methodologies.

**Figure 4:**
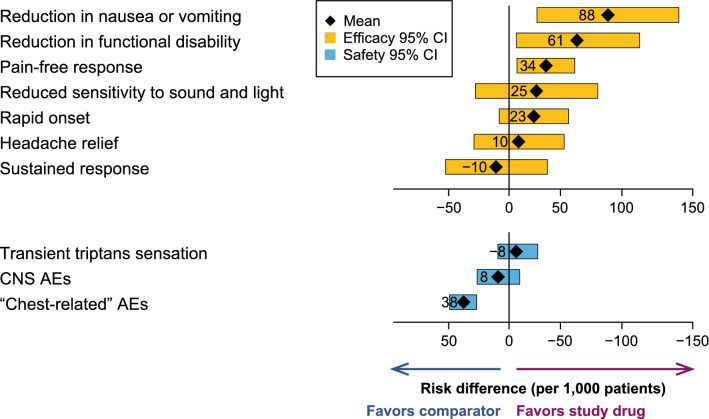
Forest plot. *AE* Adverse event, *CI* confidence interval, *CNS* central nervous system. Content is fictive and for illustrative purposes only. Figure available at: https://protectbenefitrisk.eu/dotchartspatients.html.

Forest plots (Fig. 4) are commonly used in the pharmaceutical industry as a means of displaying pooled scientific data from a clinical development program, using risk ratios for Key Clinical Benefits and Key Safety Risks [10, 26]. Our view is that these plots are fit-for-purpose for submission use in situations where the overall BR is reasonably positive and straightforward. However, forest plots may not contain sufficient context or detail to allow for more nuanced medical judgments for more complex BR scenarios.

#### Selected Fully Quantitative Methodologies

Of the various fully quantitative BR analyses, we believe that Number Needed analysis and MCDA have the greatest level of health authority recognition, particularly for use in challenging or borderline BR scenarios [2, 24, 29]. Our view is that fully quantitative methodologies require the most attention towards the concepts of detailed numerical weighting and avoiding overlap between the Key Clinical Benefits and Key Safety Risks, as these methodologies have a greater reliance on precise calculations.

Number Needed analyses (Table 4) are most useful when it is appropriate to compare a single Key Clinical Benefit with a single Key Safety Risk for a given product. Number Needed analyses are also performed to compare multiple products (e.g., NNT for drug A vs NNT for drug B). When considering such an analysis, it is important to compare like with like. For example, comparing a clinical benefit of myocardial infarction prevention with a safety risk of increased stroke is appropriate as they are similar events (major adverse cardiology events). The need to compare like with like may be considered a form of implicit weighting.

**Table 4:** NNT and NNH.

Outcome	Drug X			Placebo			NNT or NNH (95% CI) vs placebo
	*n*	*N*	%	*n*	*N*	%	
Efficacy							NNT
Response rate	143	177	80.8	226	342	66.1	7 (5, 14)
Safety							NNH
Discontinuations due to AEs	7	206	2.2	10	432	2.3	93 (− ∞, − 67) ∪ (22, + ∞)

Example of NNT and NNH. Table adapted from Citrome et al. [33]

*AE* adverse event, *CI* confidence interval, *n* number of patients with each respective outcome, *N* number of patients in the analysis treatment group, *NNH* number needed to harm, *NNT* number needed to treat. *∞* infinity, *∪* union

MCDA is a powerful tool when there are several (“multiple criteria”) Key Clinical Benefits and Key Safety Risks of a disparate nature. MCDA produces discrete numerical values for each Key Clinical Benefit and Key Safety Risk. At the most fundamental conceptual level, the discrete numerical values are derived as follows: MCDA numerical value = medical importance (as derived by a detailed weighting exercise) × frequency (observed or estimated). Weighting is the critical part of the MCDA methodology. Weighting performed by internal stakeholders can be of value; however, weighting performed by objective external key opinion leaders, subject matter experts, or patient advocacy groups may be advisable in certain situations [11, 23].

**Figure 5:**
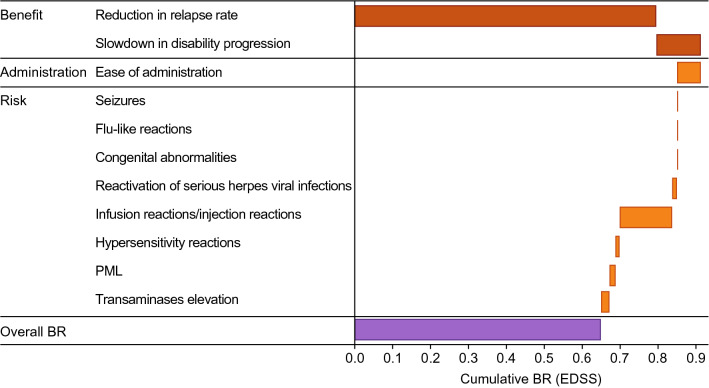
MCDA results. *BR* Benefit-risk, *EDSS* expanded disability status scale, *PML* progressive multifocal leukoencephalopathy. Content is fictive and for illustrative purposes only. Figure available at: https://protectbenefitrisk.eu/waterfallplot.html.

Discrete numerical values calculated via MCDA can be presented using various types of bar graphs (Fig. 5). To assess a favorable net clinical benefit, the additive sum of the numerical values for Key Clinical Benefits should exceed the additive sum of the numerical values for Key Safety Risks. Notably, the final derivation of MCDA is more operationally complex than indicated here and may require detailed sensitivity analyses; thus, careful consultation with biostatistics expertise, is required. While we recognize MCDA is not appropriate for the majority of products—it is complex and can be controversial—we have come to appreciate the way this methodology forces clear (and numerical) transparency regarding a team’s position on BR. We also appreciate that while this is arguably a more quantitative approach than other techniques, it remains underpinned by subjective judgement regarding the relative importance of benefits and risks, and as such may be viewed with a degree of scepticism in some quarters.

## Discussion

The production of robust and transparent sBR analyses for medicinal products is increasingly recognized as a means to better understand whether a product’s benefits outweigh its risks, and to maximize their value to prescribers and patients [11]. Such BR analyses also represents a social imperative in regard to building trust with regulators and patients, and protecting the overall public health. The field of formal and structured BR assessments is still relatively new (since the 1990s), and while increasing clarity surrounding BR has been achieved over the last 20 + years, there is still a comparative lack of consistent operating detail in terms of best practice for individual sponsors and health authorities.

Here, our intention is to promote a dialogue between the industry and health authorities regarding optimal definitions, principles, and frameworks for BR assessments for medicinal products. We define and emphasize the concepts of Key Clinical Benefits and Key Safety Risks as the foundation for BR analysis. Furthermore, we define and foundationally employ the concepts of structured BR and a Core Company BR position as the key elements for our BR framework at AstraZeneca. We have provided a simple 3-point definition for what a BR analysis is, we provide criteria to define Key Safety Risks, along with an emphasis on the weighting of Key Clinical Benefits and Key Safety Risks, and a focus on any surrounding uncertainties. Additionally, we clarify existing definitions from the IMI-PROTECT initiative [9, 30] to differentiate descriptive, semi-quantitative, and fully quantitative BR methodologies. By melding these various definitions and concepts together, we present an organically evolving sBR framework beginning from FTIH use through to the submission process, which we consider operationally fit-for-purpose, as teams are incrementally exposed to a variety of BR methodologies most in favor with health authorities.

A potential weakness of our approach is that we have as yet no formal metrics or performance indicators regarding this new process. Another challenge we have already observed is that our milestone-based recommendations for specific BR methodologies can be more difficult to apply to accelerated timeline programs where distinctions between traditional phase milestones become blurred. Furthermore, while we believe sBR brings value when done in early phase, it is important to not overwork the process when data are limited. In addition, we have not yet incorporated a formal patient-centric measure into our BR framework, although we anticipate this will be of increasing importance in the future [1, 31, 32]. Finally, it can be argued that there is no one-size-fits-all approach to BR in the pharmaceutical industry and that one’s particular choices for frameworks and methodologies may not matter so long as they’re done via a structured, systematic approach.

Also, as noted above, there are a large number of BR methodologies in existence, and it is possible that by our focused approach in recommending only a handful of these methodologies we could be missing the value of those we have not selected. We also recognize that, despite our argument for the value case of sBR, there likely remain pharmaceutical sponsors and regulatory authorities that believe the legacy approach to relying primarily on descriptive BR is sufficient and may not agree with our proposal that semi-quantitative BR assessments represent a base case standard in most instances. In this regard, we look forward to the anticipated recommendations due from the CIOMS XII working group, whose focus is on quantitative and qualitative approaches to the evaluation of BR.

In any event, given the increasing priority on more formal analysis of BR in the pharmaceutical industry and among health authorities, we aim to contribute to the conversation among sponsors and health authorities regarding the practical application of best practice in the BR field.

## Conclusion

AstraZeneca has reorganized their organizational and procedural approach for overall BR assessments by defining and emphasizing the concept of sBR, which we believe represents an appropriate foundational principle for both producers and customers of pharmaceutical assessments. In addition, we have provided a simple definition to demystify the production of BR assessments, defined a tripartite classification of methodological approaches to BR (i.e., descriptive, semi-quantitative, and quantitative), and established a suggested hierarchical methodologic progression for the assessment of BR for medical products at key timepoints during the development process. By presenting our framework, we wish to stimulate productive conversation between industry peers and health authorities regarding best practice in the BR field. This paper may also help facilitate the pragmatic implementation of sBR methodologies for organizations without an established framework for such assessments.

## Data Availability

There are no data presented in this manuscript, however further information on any of the content can be requested from the corresponding author.

## Appendix 1. Instructions for Using the Modified FDA BR Framework

Keep the table as concise as possible, aiming for a total size of 1–2 pages.

**Table:**

	Overview and evidence	Uncertainties and implications
Analysis of condition		
Current treatment options		
Potential clinical benefits		
Potential safety risks		

### Analysis of Condition

What is the precise target indication/population and why was it chosen? Characterize the unmet need. How well is the population understood? What is the estimated size of the population? Will there be any subpopulations of special interest? What are the key comorbidities and disease-related symptoms? What is the expected age of the population? Will you include children or the elderly? What is the time course and prognosis? Is accelerated development or approval anticipated? Are there any factors known to be of particular importance to patient groups? Is this a chronic or acute condition? We need to understand the severity of the condition being treated to understand the level of risk that is acceptable, i.e., life-threatening vs prophylactic.

### Current Treatment Options

What is the standard of care treatment, and what are the other treatment options, including their degree of use and market share? What is their response rate and clinical effect/importance? Are there known issues associated with compliance, ease of use, formulation, subgroups, etc.? What is the upcoming competitive landscape, including developmental products? What are the key clinical use factors? What is the safety profile and tolerability of the current standard of care? Is there any unmet medical need in the context of safety?

### Potential Clinical Benefits

What is the expected potential efficacy of the drug and how do you measure it? What is the precise and evidence-based justification for potential efficacy? Are there any important uncertainties regarding therapeutic effect? What are the basics of the mechanism of action (MoA) and pharmacokinetics-pharmacodynamics, and how well are they understood? Is the MoA recognized to be effective in the target indication? Is the efficacy biomarker driven? How validated and accepted are preclinical models of efficacy, if any? What is the anticipated clinical importance of the benefit and how will it be characterized? What will be the key efficacy objectives and endpoints of the development? Acknowledging that certain benefits may be best measured by Patient Reported Outcomes (PROs), please justify your choice to include/exclude these measures.

### Potential Safety Risks

What is the summary of important preclinical findings from SARB, including color grade? Are there any dose-limiting toxicities affecting human dosing and safety margins? What is the potential for on and off target effects? Does the MoA have any known or theoretical association with safety risks or class effects? Do potential competitors have any important safety issues highlighted by health authorities, adverse reactions, identified risks prominent in labeling, or requiring special risk management activities? Can any Key Safety Risks be identified and are any special risk management measures needed, e.g., toxicity management guidelines? Are the risks manageable by a general dose modification scheme? Do you need any specific treatment to prevent or treat any of the Key Safety Risks (e.g., premedication or antidotes)? Do you need special monitoring for the individual risks and how feasible is special monitoring in the clinical trial setting?

## Appendix 2. Brief Primer: Fully Quantitative BR Analysis in the Pharmaceutical Industry

Weighting the relative importance of clinical benefits and safety risks for MCDA.

### Context Regrading BR Analysis

Formal assessments of BR are now an expected part of the drug approval process. Sponsors develop these assessments, which in turn are critiqued by health authorities. The most basic acceptable form of BR analysis is called a value tree, which is a list of the product’s Key Clinical Benefits and Key Safety Risks. It is important to keep the number of Key Clinical Benefits and Key Safety Risks as concise as possible to focus attention on the most important attributes of the given product.

Increasingly, health authorities are emphasizing the need for more quantitative assessments of BR, as opposed to simple lists of benefits and risks. This is particularly true for more complex or challenging BR scenarios. The most rudimentary quantitative BR method is Number Needed to Treat (NNT) compared with Number Needed to Harm (NNH). This can be a powerful method; however, its use is limited to like with like situations, where a single important Key Clinical Benefit can be easily compared to a single important Key Safety Risk. For example, comparing a Key Clinical Benefit of reduction in myocardial infarction to a Key Safety Risk of increases in intracranial bleeding.

### Context Regarding MCDA

It is common to have a BR scenario where several Key Clinical Benefits (sometimes of varying types) need to be compared to multiple Key Safety Risks (typically of various types). These are no longer like with like (apple with apple) comparisons; instead, they are “fruit salad-to-fruit salad” comparisons. In these more complex BR scenarios, the use of MCDA is increasingly recognized by health authorities as a potentially useful tool. Most people use the MCDA concept every day without recognizing it as such, as we make intuitive decisions taking into account a variety of competing interests (i.e., multiple criteria).

When the stakes are high, such as a potential drug approval, it can in certain instances be useful to construct more formal and transparent models of MCDA instead of relying on simple intuition. In essence, the objective in such an MCDA is to methodically determine a single numerical value for each Key Clinical Benefit and each Key Safety Risk. This single numerical value for each benefit and risk is referred to as a ‘BR score’. If the arithmetic sum of the BR scores for a product’s Key Clinical Benefits outweighs the arithmetic sum of the BR scores for that product’s Key Safety Risks, this is considered a favorable BR profile (and vice versa).

### Context Regarding the Concept of BR Scores

Before proceeding further, it is important to highlight that these BR scores represent arbitrary numerical values. For example, a BR score for a Key Clinical Benefit could be ‘1’, ‘100’, or ‘1000’. The actual number does not matter- again, it is arbitrary. What matters is how the BR score for a Key Clinical Benefit compares relative to the BR score for a Key Safety Risk. For example, a Key Clinical Benefit with a BR score of ‘10’ has a more favorable BR profile compared with a Key Safety Risk with a BR score of ‘5’ (i.e. because 10 is greater than 5). To establish the point that these are arbitrary values, we can express the same favorable BR profile as ‘100’ vs ‘50’ or ‘1000’ vs ‘500’. What matters is the relative values of these numbers (i.e., in this particular example, the benefit is 2 times greater than the risk).

The BR score is calculated as follows: The observed (or estimated) frequency of a benefit (or risk) × the medical importance of that benefit (or risk). However, the actual operation of this conceptually simple formula requires some explanation.

### Context on the Actual Calculation of BR Scores

Clearly, if one is to arrive at a single numerical BR score, it is necessary to multiply a number representing frequency by a number representing medical importance. We address each of these topics below.

#### Frequency

At first, deriving a number for frequency seems easy. Key Clinical Benefits are often expressed as responder rates, while Key Safety Risks are often expressed as incidence rates per unit time (both are numbers). Where it gets a little tricky is that these are different types of numbers. Thus, it is not appropriate to directly compare, say, a 33% responder rate with a 10% per patient year adverse event rate, as they are different types of rates. To overcome this, a normalization exercise is undertaken using a so-called “value function” where different types of rates, incidences, etc. are normalized into numerical values representing frequency, which are more appropriate to compare with one another.

#### Medical Importance

It is normal to question how one might apply a numerical value to the concept of medical importance. This is accomplished via a weighting exercise, which involves medical assessors making subjective value judgments, comparing the relative importance of Key Clinical Benefits and Key Safety Risks. In other words, a discrete numerical value is attached to each Key Clinical Benefit and Key Safety Risk, which represents the personal opinion of the medical assessor. Further details are included below.

### Justification to Perform Weighting Assessments for Key Clinical Benefits and Key Safety Risks

It is normal to question the appropriateness of individual medical assessors to use their personal opinion to concretely quantify the medical importance of any topic. However, these subjective assessments are made implicitly every time a sponsor submits a product for approval or when a regulator decides to approve that product for use. Each of us are making implicit value judgments every day, whether in the pharmaceutical industry or in our personal lives.

MCDA takes these implicit judgments and makes them more transparent by making them explicit. By transparently committing medical judgments to paper, we open these judgments to more effective peer review. This concept is at the heart of MCDA.

### Methodology to Perform Weighting Assessments for MCDA

#### Arbitrary Values Used for Relativistic Comparisons

The end goal of the MCDA weighting process is that we have a discrete numerical value characterizing the relative medical importance of each Key Clinical Benefit and Key Safety Risk. As previously noted, these will be arbitrary numerical values. For example, the medical importance of a Key Clinical Benefit may be ‘1000’, which is twice as medically important as a Key Safety Risk with a value of ‘500’.

#### Establishing an Anchor for Weighting Comparisons

To begin a weighting exercise for Key Clinical Benefits and Key Safety Risks, we need an ‘anchor’. This term refers to the starting point to which all other benefits and risks are compared. The anchor will be the single most important Key Clinical Benefit; most typically, the primary endpoint of the pivotal efficacy studies. The anchoring Key Clinical Benefit is assigned an arbitrary medical importance value of ‘1000’.

The next step is to add in the weighting for any additional Key Clinical Benefits, which will have a medical importance value less than ‘1000’. In the example shown below, Key Clinical Benefit B is 60% as important as Key Clinical Benefit A.

The final step involves adding in relative weights for the Key Safety Risks. Internal experience shows that this can be a confusing step for many assessors. We have found it helps to start with the following exercise: Is one patient with Key Safety Risk C more or less important than one patient with Key Clinical Benefit A? In other words, put yourself in the shoes of an FDA medical reviewer.

In this simplified example, the product under review was studied in two patients. One patient reported Key Clinical Benefit A with no side effects. The second patient reported Key Safety Risk C with no benefit. Would you approve the product? Here, Key Safety Risk C represents a serious safety issue frequently associated with mortality. Let’s say that the medical importance of Key Safety Risk C is 10 × greater than the medical importance of Key Clinical Benefit A, which is reflected with a weighting score of ‘10,000’ for Key Safety Risk C. In this case, the product would not be approved (i.e., assuming equal frequencies for both the benefit and the risk).

Let’s continue the exercise with a few additional Key Safety Risks. Key Safety Risk D is a serious safety issue; while not associated with mortality, it is frequently associated with hospitalization and disability. We will say it is half as important as Key Safety Risk C, thus giving Key Safety Risk D a medical importance value of ‘5000’. Key Safety Risk E is a non-serious tolerability issue, which is easily treated by lowering the dose of the product, and so is much less important than Key Clinical Benefit A, with a medical importance score of only ‘50’. Both of these examples are illustrated below.
